# Suppression of Light-Induced Oxidative Stress in the Retina by Mitochondria-Targeted Antioxidant

**DOI:** 10.3390/antiox8010003

**Published:** 2018-12-21

**Authors:** Viktoriia E. Baksheeva, Veronika V. Tiulina, Natalia K. Tikhomirova, Olga S. Gancharova, Sergey V. Komarov, Pavel P. Philippov, Andrey A. Zamyatnin, Ivan I. Senin, Evgeni Yu. Zernii

**Affiliations:** 1 Belozersky Institute of Physico-Chemical Biology, Lomonosov Moscow State University, Moscow 119992, Russia; vbaksheeva@belozersky.msu.ru (V.E.B.); tyulina_nika@list.ru (V.V.T.); tikhomir@belozersky.msu.ru (N.K.T.); olgancharova@belozersky.msu.ru (O.S.G.); ppph@belozersky.msu.ru (P.P.P.); zamyat@genebee.msu.ru (A.A.Z.J.); senin@belozersky.msu.ru (I.I.S.); 2 Institute for Regenerative Medicine, Sechenov First Moscow State Medical University, Moscow 119991, Russia; 3 Department of Biology and Pathology of Domestic, Laboratory and Exotic Animals, Skryabin Moscow State Academy of Veterinary Medicine and Biotechnology, Moscow 109472, Russia; skomarov1977@mail.ru; 4 Institute of Molecular Medicine, Sechenov First Moscow State Medical University, Moscow 119991, Russia

**Keywords:** light-induced retinal damage, age-related macular degeneration, oxidative stress, antioxidant activity, superoxide dismutase, glutathione peroxidase, mitochondria-targeted antioxidant, SkQ1, visual arrestin, disulfide dimerization of proteins

## Abstract

Light-induced oxidation of lipids and proteins provokes retinal injuries and results in progression of degenerative retinal diseases, such as, for instance, iatrogenic photic maculopathies. Having accumulated over years retinal injuries contribute to development of age-related macular degeneration (AMD). Antioxidant treatment is regarded as a promising approach to protecting the retina from light damage and AMD. Here, we examine oxidative processes induced in rabbit retina by excessive light illumination with or without premedication using mitochondria-targeted antioxidant SkQ1 (10-(6’-plastoquinonyl)decyltriphenyl-phosphonium). The retinal extracts obtained from animals euthanized within 1–7 days post exposure were analyzed for H_2_O_2_, malondialdehyde (MDA), total antioxidant activity (AOA), and activities of glutathione peroxidase (GPx) and superoxide dismutase (SOD) using colorimetric and luminescence assays. Oxidation of visual arrestin was monitored by immunoblotting. The light exposure induced lipid peroxidation and H_2_O_2_ accumulation in the retinal cells. Unexpectedly, it prominently upregulated AOA in retinal extracts although SOD and GPx activities were compromised. These alterations were accompanied by accumulation of disulfide dimers of arrestin revealing oxidative stress in the photoreceptors. Premedication of the eyes with SkQ1 accelerated normalization of H_2_O_2_ levels and redox-status of lipids and proteins, contemporarily enhancing AOA and, likely, sustaining normal activity of GPx. Thus, SkQ1 protects the retina from light-induced oxidative stress and could be employed to suppress oxidative damage of proteins and lipids contributing to AMD.

## 1. Introduction

The mammalian retina is highly vulnerable to oxidative stress. Indeed, photoreceptor and retinal pigment epithelium (RPE) cells generate high levels of reactive oxygen species (ROS) due to a number of factors. First, photoreceptors are constantly exposed to light and contain a number of different photosensitizer molecules. Second, they metabolize and function under high oxygen conditions: the retina is characterized by extremely high oxygen consumption [1]. Third, membranes of photoreceptor discs are enriched in polyunsaturated fatty acids that are especially susceptible to oxidative damage [2]. The recycling of photoreceptor discs suffering from oxidative damage is performed via their phagocytosis by RPE and oxidative damage to photoreceptors induces intense accumulation of ROS in pigment epithelium [3]. These processes impose a great risk on the retina, especially in older people, as retinal neurons and RPE cells become especially susceptible to oxidative damage in the aging tissue [4,5].

Oxidative damage to the retina is commonly induced by prolonged eye exposure to natural or anthropogenic light sources resulting in progression of light retinopathy (light/photic maculopathy) [6,7,8,9,10]. Having accumulated over years, light-induced injuries were suggested to provoke age-related macular degeneration (AMD), the leading cause of vision deterioration and blindness in the elderly [11,12]. Consistently, there are pronounced similarities between oxidative retinal damage in experimental animals exposed to bright light, and in late-stage AMD patients [13,14]. Indeed, the essential features of human dry (non-neovascular) AMD can be reproduced in the models of light-induced retinal damage (LIRD) [15,16,17,18]. Our previous histological studies revealed that retinas of the animals exposed to various doses of light illumination exhibit such hallmarks of AMD as photoreceptor apoptosis, pigment epithelial cell migration into the neural retina, disturbance in blood-retinal barrier, and inflammatory infiltration [19,20]. The studies using these models may provide important information about general mechanisms of retinal photic damage underlying AMD and other degenerative retinal diseases. The latter include, for instance, iatrogenic light-induced retinopathies caused by illumination from various devices such as slit lamps, indirect ophthalmoscopes, fiber optic endo-illuminators and light sources of operative microscopes [21,22]. Current advances in ophthalmic surgery and eye care increased the incidence of iatrogenic photochemical damage to the retina, which thereby represents another relevant problem of modern ophthalmology [9,10,21].

An urgent task is identifying approaches to the prevention and treatment of AMD and light-induced retinopathies, which requires understanding of mechanisms underlying oxidative damage of the retinal cells. One of the possible mechanisms of photoreceptor cell death induced by light is mitochondria-mediated apoptosis [23]. Mitochondria serve as the main target of calcium toxicity, initiated by the influx of calcium ions into the photoreceptor cells in response to light damage to their outer segments [24]. Light-induced degeneration of RPE cells could also be mediated by mitochondria. For instance, the function of these organelles is impaired by high levels of all-*trans*-retinal, the product of rhodopsin, excessively accumulating in the RPE in response to intense illumination [25]. Another proposed mechanism of the RPE degeneration is oxidative damage to mitochondrial DNA [26]. Considering these findings, antioxidant treatment could be regarded as a promising approach to protecting the retina from light-induced damage. 

In general, conventional antioxidants have low bioavailability especially in the retina, which is isolated from the bloodstream [27,28,29]. Consistently, there is mixed evidence on the effectiveness of dietary uptake of such antioxidants for prevention and therapy of common ophthalmological conditions [30]. Yet, during the last decade a new class of mitochondria-targeted antioxidants has emerged as a perspective response to age-related diseases. One of these compounds is SkQ1 (10-(6’-plastoquinonyl)decyltriphenyl-phosphonium), a plastoquinol derivative modified by a lipophilic cation that allows penetration of the drug into the mitochondria inner membrane and its accumulation in mitochondrial matrix [31]. SkQ1 possesses high bioavailability upon topical administration in the form of eye drops: nanomolar concentrations of SkQ1 were detected in rabbit retina after instillation of SkQ1-containing eye drops by a liquid chromatography-tandem mass spectrometry (LC-MS/MS) method (Nevinitsyna et al, 2019, manuscript in preparation). Therefore, it exhibits prominent protector activity towards different eye tissues when used in nanomolar to low micromolar concentrations. Due to these features SkQ1 demonstrated efficacy in prophylactics and therapy of glaucoma and uveitis as well as corneal lesions and dry eye syndrome of different etiologies [32,33,34]. 

Recently, using a rat model of light-induced retinal degeneration we demonstrated that SkQ1 exhibits prominent protective effect towards photoreceptors and RPE cells [19]. In the current study we addressed oxidative processes in the retina during 1 week after exposure of the rabbit eyes to intense light with or without premedication using SkQ1. We demonstrated that the illumination enhances lipid peroxidation and hydrogen peroxide levels in the retinal cells, which is associated with decrease in activity of antioxidant defense enzymes, superoxide dismutase (SOD), and glutathione peroxidase (GPx). Unexpectedly, we observed an increase in total antioxidant activity (AOA) in the retina suggesting the existence of mechanisms of its upregulation in response to light/oxidative conditions. These alterations were accompanied by disulfide dimerization of visual arrestin, indicating development of oxidative stress in photoreceptor cells. Premedication of the retinas with SkQ1 was shown to accelerate normalization of hydrogen peroxide levels and redox-status of lipids and photoreceptor proteins contemporarily enhancing AOA and sustaining normal activity of GPx. Our data indicate that SkQ1 protects the retina from light-induced oxidative stress and therefore it could be employed to suppress oxidative damage of proteins and lipids contributing to ocular pathology, specifically AMD.

## 2. Materials and Methods

### 2.1. Materials

SkQ1 (10-(6′-plastoquinonyl)-decyltriphenylphosphonium) was provided by the Institute of Mitoengineering of Moscow State University (Moscow, Russia). Phosphate buffer saline (PBS) was from Thermo Fisher Scientific (Waltham, MA, USA). Tiletamine and zolazepam were from Virbac (Carros, France). Xylazine hydrochloride was from Nita-Farm (Reutov, Russia). Reagents for histological examination were from Biovitrum (St. Petersburg, Russia). Trolox (6-hydroxy-2,5,7,8-tetramethylchroman-2-carboxylic acid), superoxide dismutase and malondialdehyde assay kits were from Sigma-Aldrich (St. Louise, MO, USA). Glutathione peroxidase assay kit was from Randox (Crumlin, UK). Polyclonal anti-visual arrestin antibodies were previously produced by animal immunization and affinity purification from hyperimmune serum [35]. Mouse monoclonal anti-visual arrestin antibodies were from Santa Cruz Biotechnology (Dallas, TX, USA) (sc-271159). Secondary antibodies were from Jackson ImmunoResearch (Cambridge, UK) (115-035-003 and 111-035-003). Supplies for Western blotting were from (Bio-Rad Laboratories Inc., Hercules, USA). Other reagents and supplies were from Sigma-Aldrich, Amresco (Cleveland, OH, USA), and Serva (Heidelberg, Germany). All buffers were prepared using ultrapure deionized water.

### 2.2. Animals and Ethics Statement

The study involved 87 pigmented male rabbits (6 months old, 2.3 to 3 kg) purchased from a certified farm (Krolinfo, Moscow, Russia). Rabbits were chosen as model animals due to similarities in ocular morphology and biochemistry between human and rabbit eyes [6]. The rabbits were housed individually in 795 × 745 × 1776 mm cages at a 12 h light-dark cycle at a temperature of 22–25 °C and humidity of 55–60% with access to maintenance rabbit food and water ad libitum. The health status of the animals was monitored daily and no adverse events were observed. Before the experiments, the animals were housed as described above for one week for acclimatization. All experiments were performed under general anesthesia anesthesia induced with intramuscular injection of 1:2 mixture of 50 mg/mL tiletamine/zolazepam and 20 mg/mL xylazine hydrochloride. After the completion of the experiments the animals were humanely euthanized by an overdose of the anesthetic. Enucleating of the eyeballs and harvesting of the retinas were performed postmortem. The treatment of the animals was performed according to the 8th edition “Guide for the Care and Use of Laboratory Animals” of the National Research Council and “Statement for the Use of Animals in Ophthalmic and Visual Research” of The Association for Research in Vision and Ophthalmology (ARVO). The protocol was approved by the Belozersky Institute of Physico-chemical Biology Animal Care and Use Committee (Protocol number 1/2016). 

### 2.3. Experimental Model

The experiments were performed using a single-blind method. 18 rabbits (groups 1–3, 6 animals per group) were employed in a histological study of LIRD. Group 1 contained intact animals, groups 2 and 3 were illuminated with bright visible light. One hour before the illumination the animals were premedicated by 6 subsequent conjunctival instillations (1 instillation per 10 min) of 50 μL of either 7.5 μM SkQ1 in vehicle solution (7 mM PBS, pH 7.4, 0.0001% benzalkonium chloride) or placebo (vehicle solution) as described in [34]. After that, the animals were placed in a restraining device, anesthetized as described in Section 2.2 and illuminated with a halogen lamp (30,000 lx, 0.15 W/cm^2^) from a distance of 40 cm for 3 h. Additional injections of the anesthetic were performed in the course of anesthesia to uphold continuous narcotic sleep. The animals were sacrificed after keeping under normal conditions described above for 7 days and their eyes were subjected to fixation and histological analysis.

A number of 42 rabbits was separated into 7 groups of six animals: intact animals (group 4); illuminated animals premedicated with SkQ1 (groups 5–7); illuminated animals premedicated with placebo (groups 8–10). The animals were sacrificed after 1 (groups 5, 8), 3 (groups 6, 9) or 7 days (groups 7, 10) and their retinas were used for biochemical measurements. 

A number of 27 rabbits were separated into 9 groups of 3 animals and their retinal extracts were used for Western blotting analysis of arrestin dimerization: group 11 contained intact animals, groups 12–15 received SkQ1 and groups 16–19 received placebo before illumination under the same conditions as the previous groups. The rabbits were sacrificed 3 h (groups 12, 16), 1 day (groups 13, 17), 3 days (groups 14, 18), and 7 days (groups 15, 19) after the illumination.

### 2.4. Measurements of Outer Nuclear Layer Thickness

The eyeballs of the animals were enucleated immediately post-mortem and fixed in 4% formalin in PBS (pH 7.4). Fixed eyes were sectioned through their vertical diameter, yielding material for histological analysis that contained the central and peripheral retinal areas. The half-eyes were subjected to routine histological procedure including dehydration in a graded ethanol series and clearing in xylene in an automatic tissue processor. Samples were embedded in paraffin taking into account the sample orientation. Four-micron-thick sagittal cross-sections through central areas of posterior sectors were obtained along the eyes vertical meridian from the paraffin blocks. The sections were mounted on Menzel–Gläser glass slides (Thermo Fisher Scientific), hydrated, and stained with Carazzi’s hematoxylin and eosin Y. Histological preparations were examined using a Leica DM4000 (Leica, Wetzlar, Germany). Microphotographs were obtained using a high-resolution digital camera Leica DFC420 (Leica). Viewing and processing of microphotographs and addition of scales were performed using AxioVision 8.0 (Carl Zeiss, Oberkochen, Germany) and Adobe Photoshop CS3 software (Adobe Systems Inc., San Jose, CA, USA).

The outer nuclear layer (ONL) thickness was measured by light microscopy on retinal microphotographs of hematoxylin-eosin stained sections. Images of slides were captured digitally with standardized microscope and camera settings. In order to standardize all tissue sample locations measurements were made at two equidistant (1000 μM from the optic nerve head) foci in both the inferior and superior retinal hemispheres. Measurements were made by personnel unaware of the study groups.

### 2.5. Retinal Samples 

The retinas from the intact and illuminated rabbit eyes were isolated in a dim red light at 4 °C immediately after the scarification. Each isolated retina was homogenized for 1 minute on ice in 0.3 mL of 50 mM Tris-HCl, 100 mM NaCl buffer (pH 7.5) using HG-15A 27,000 rpm homogenizer (Witeg Labortechnik, Wertheim, Germany) and the resulting fraction was centrifuged at 24,000× *g* (16,000 rpm) for 20 min at 4 °C. The supernatants (retinal extracts) were stored at −70 °C until the further studies. The pellets were homogenized in the MDA lysis buffer (Sigma-Aldrich) and the resulting fractions (retinal homogenates) were used for lipid peroxidation measurements.

### 2.6. Total Protein Concentration

Total protein content in retinal extracts was measured by the bicinchoninic acid (BCA) method using a BCA protein assay kit (Thermo Fisher Scientific) following the protocol provided by the manufacturer.

### 2.7. Malondialdehyde Concentration

Lipid peroxidation level was estimated from malondialdehyde concentration in retinal homogenates via thiobarbituric acid assay, using commercially available kit (Sigma-Aldrich) according to the manufacturer’s instructions. 

### 2.8. Hydrogen Peroxide Concentration 

H_2_O_2_ levels were determined using a method previously described by Erel et al. [36]. H_2_O_2_ present in retinal extract oxidizes the Fe^2+^/o-dianisidine complex to Fe^3+^, which produces a colored complex with xylenol orange. The color intensity was measured spectrophotometrically at 560 nm.

### 2.9. Total Antioxidant Activity

Total antioxidant activity of retinal extracts was analyzed by means of hemoglobin/H_2_O_2_/luminol assay according to the standard procedure [37] with modifications described previously [34,38,39]. Briefly, 1–8 μM Trolox or retinal extract (4 mg/mL of total protein) was added to the reaction mixture, containing 0.01 mM luminol and 0.5 mM hemoglobin in PBS. The reaction was started with addition of H_2_O_2_ to a final concentration of 6 μM and brief vortexing of the sample. Chemiluminescence was registered each 1 s for 10 min using Glomax–Multi Detection System luminometer (Promega, Madison, WI, USA). Antioxidant activity of the samples was expressed in Trolox equivalent.

### 2.10. Antioxidant Enzymes Activity

The activity of superoxide dismutase (SOD) and glutathione peroxidase (GPx) in retinal extracts was evaluated using commercially available kits in accordance with the manufacturer’s instructions. Intensity of the colorimetric reactions was determined using Synergy H4 Hybrid Reader (Biotek, Winooski, VT, USA) or Ultrospec 1000 (Pharmacia, Kearny, NJ, USA). 

### 2.11. Content of Disulfide Dimers of Arrestin 

The content of disulfide dimers of visual arrestin in the retinal extracts was determined by non-reducing Western blotting as described in [20]. The arrestin was visualized using polyclonal (monospecific) antibodies obtained previously (1:10,000 in Tris buffer saline with 0.05% Tween-20 (TBST)) [35]. Alternatively, visual arrestin (C-1) mouse monoclonal IgG1 antibodies were used (1:5000 in TBST). Horseradish peroxidase-conjugated secondary antibodies were applied in dilution, recommended by the manufacturer (1:1000 in TBST). All samples were normalized by total protein content (80 μg per lane), as determined by BCA analysis. The protein bands were visualized in ChemiDoc™ XRS+ gel documentation system (Bio-Rad) using the enhanced chemiluminescence (ECL) kit (Bio-Rad). The weight fractions of arrestin forms were estimated from the immunoblots by densitometric scanning of the protein bands and the data analysis using GelAnalyzer v.2010a software [40]. 

### 2.12. Statistical Analysis

The data were analyzed by the mean standard error (SE) method. Mean scores, SE, and statistical significance were calculated with SigmaPlot 11 (SYSTAT Software, San Jose, CA, USA). Statistical significance was evaluated with the Mann–Whitney U test. The probability of 0.05 was considered significant.

## 3. Results

### 3.1. Morphological State of Bright Light-Exposed Retina: Thickness of Outer Nuclear Layer

Light-induced oxidative damage in the retina was simulated in the following animal model. Both eyes of restrained pigmented rabbits were exposed to short-term (3 h) illumination by visual light of high intensity (halogen lamp, 30,000 lx; 0.15 W/cm^2^). Prior to the illumination, the animals from experimental groups were premedicated with six subsequent conjunctival instillations (1 instillation per 10 min) of 50 μL of 7.5 μM SkQ1 in vehicle solution (7 mM PBS, pH 7.4, 0.0001% benzalkonium chloride) and are referred to as “premedicated”. The “control” groups received instillations of placebo (vehicle solution) administered in the same manner. The rabbits were sacrificed and their eyes were collected 7 days after the illumination and subjected to histological analysis. The eyes of unexposed animals were employed as a reference.

**Figure 1:**
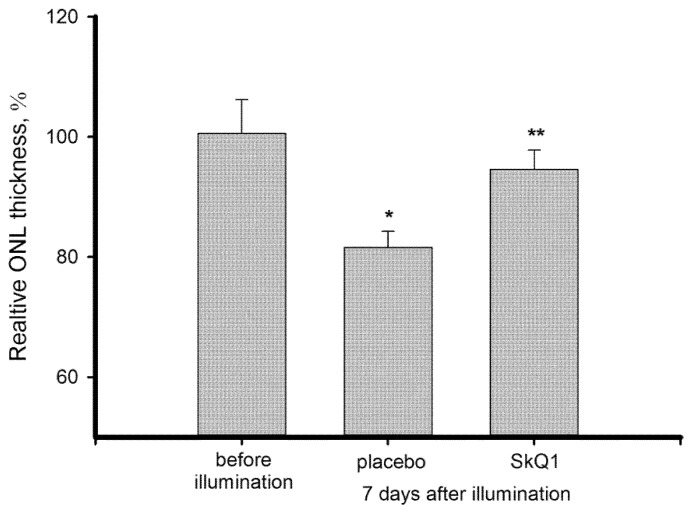
Normalized outer nuclear layer (ONL) thickness in the illuminated retinas of rabbits with or without premedication with 7.5 μM SkQ1. ONL thickness in the intact retinas is taken as 100%. Each bar represents the data obtained from six animals (12 eyes). * *p* ≤ 0.05 compared with the values measured in the intact animals; ** *p* ≤ 0.05 compared with the values measured in the control animals.

LIRD was assessed from the survival rate of photoreceptors in the illuminated retina via measurement of the mean outer nuclear layer (ONL) thickness at the reference points located at 1000 μM from the center of the optic nerve disc. It was found that 7 days after the illumination the ONL of control retinas was approximately 19% (by ~4 μM) thinner than in the intact retinas (Figure 1). Meanwhile, the ONL thickness of the premedicated animals was statistically equal to the norm. Thus, SkQ1 premedication was demonstrated to prevent light-induced photoreceptor loss.

### 3.2. Light-Induced Oxidative Stress in the Retina: Lipid Peroxidation and Hydrogen Peroxide Accumulation

**Figure 2:**
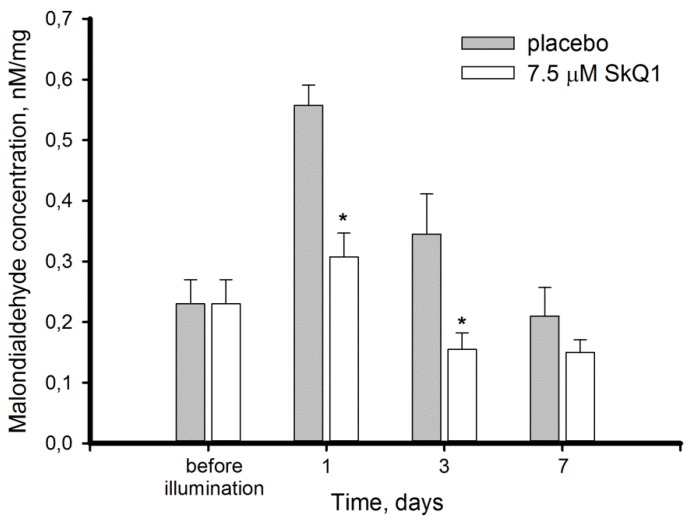
Malondialdehyde (MDA) concentration in homogenates of intact and illuminated rabbit retinas with or without premedication with 7.5 μM SkQ1. MDA concentration was determined by thiobarbituric acid assay. Each bar represents the data obtained from six animals (12 eyes). * *p* ≤ 0.05 compared with the values measured in the control animals.

Oxidative processes in the retina exposed to excessive illumination and effect of SkQ1 premedication on these processes, were assessed in the retinas obtained from the intact and illuminated rabbit eyes 1, 3, and 7 days after the exposure. The retinal specimen were homogenized in a detergent-free buffer and centrifuged, yielding membrane pellets and soluble extracts. These fractions were used to monitor manifestations of light-induced oxidative stress in membrane and cytosol of the retinal cells. In particular, the membrane fraction was analyzed for the levels of lipid peroxidation (expressed in MDA concentration), whereas the soluble extracts were assessed for hydrogen peroxide content. Without SkQ1, MDA concentration in retinal homogenate elevated 3-fold at the first day and normalized on day 7 of the post-exposure period (Figure 2). In the animals premedicated with 7.5 μM SkQ1 MDA elevation was suppressed two-fold as compared to placebo, and restoration of normal MDA level was achieved on day 3. 

**Figure 3:**
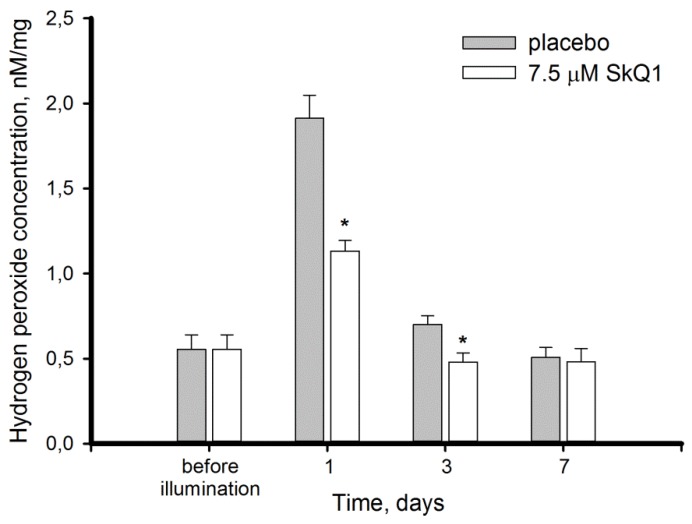
Hydrogen peroxide content in extracts of intact and illuminated rabbit retinas with or without premedication with 7.5 μM SkQ1. Hydrogen peroxide concentration was measured by Fe^2+^/o-dianisidine/xylenol orange colorimetric assay. Each bar represents the data obtained from six animals (12 eyes). * *p* ≤ 0.05 compared with the values measured in the control animals.

The MDA elevation in the control retinas was accompanied by accumulation of H_2_O_2_, which increased 4-fold within 24 h after illumination and fully normalized only after 7 days of the post-exposure period (Figure 3). Premedication with SkQ1 reduced the initial H_2_O_2_ elevation by 41% and slightly accelerated its recovery: in the premedicated group H_2_O_2_ content completely normalized after 3 days. 

Taken together, these data indicate that short-term exposure of the rabbit eyes to high-intensity illumination results in the development of pronounced oxidative stress that spontaneously resolves after 7 days of the follow-up period. Meanwhile premedication of the eyes with SkQ1 alleviates this condition and accelerates its recovery. In particular, it suppresses oxidative damage to cellular lipids, which is known to generate toxic compounds, contributing to ocular pathology.

### 3.3. Antioxidant Activity in the Bright Light-Exposed Retina: Total Antioxidant Activity and Antioxidant Enzyme Functioning

**Figure 4:**
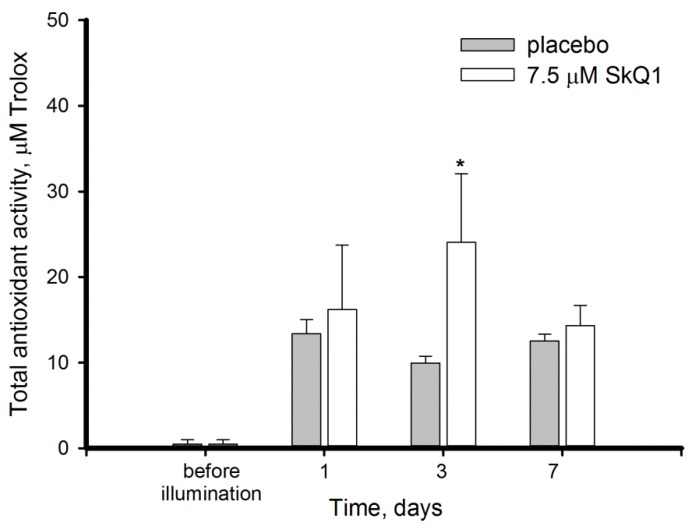
Total antioxidant activity (AOA) in extracts of intact and illuminated rabbit retinas with or without premedication with 7.5 μM SkQ1. AOA was determined by hemoglobin/H_2_O_2_/luminol assay. Each bar represents the data obtained from six animals (12 eyes). * *p* ≤ 0.05 compared with the values measured in the control animals.

Considering that light illumination induced ROS accumulation and triggered oxidative processes in the retina, we next explored the responses of its intrinsic antioxidant mechanisms to the stress conditions with or without premedication with SkQ1. First, we monitored total AOA of the retinal extracts by hemoglobin/H_2_O_2_/luminol assay that detects presumably low-molecular weight ROS scavengers, such as glutathione and ascorbate. Unexpectedly, no latency of luminol oxidation was detected in the presence of intact retina extracts indicating very low AOA, the level of which likely remained beyond the method detection limits (Figure 4). However, the illumination resulted in an increase of AOA in the retina, although it remained significantly lower than that in the other eye tissues measured by the same method [34,38]. SkQ1 had a moderately positive effect on this increase, especially on day 3, when AOA in was 2.4-fold higher than in placebo group. 

**Figure 5:**
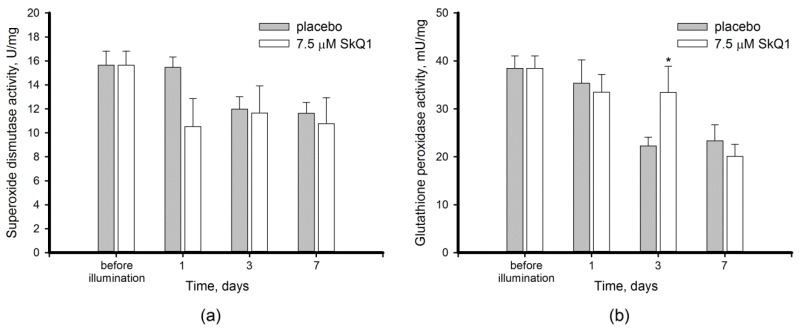
Antioxidant enzyme activity in extracts of intact and illuminated rabbit retinas with or without premedication with 7.5 μM SkQ1. (**a**) Superoxide oxidase (SOD) activity; (**b**) glutathione peroxidase (GPx) activity. Enzyme activities were determined using standard colorimetric assays as described in the Materials and Methods section. Each bar represents the data obtained from six animals (12 eyes). *p* ≤ 0.05 compared with the values measured in the control animals.

In addition to the total AOA, the activity of antioxidant enzymes was reliably changed upon the pronounced light-induced oxidative stress in the retina. Thus, in the placebo group the activity of SOD and GPx concomitantly decreased on day 3 post exposure by 21% (Figure 5a) and 57% (Figure 5b), respectively. Both enzymes remained downregulated during the next 7 days reflecting long-term response to the oxidative stress. Yet, the impacts of premedication with SkQ1 on these enzymes were small and possessed a different tendency. Thus, no statistically significant effect of the antioxidant towards SOD was found, although a slight decrease in activity of the enzyme could be noted in the premedicated animals already on the first day after illumination. By contrast GPx activity was likely sustained at normal level until day 7 of the post-exposure period. 

Overall, we revealed for the first time that intact mammalian retina possesses low AOA, which increases upon intense light exposure suggesting existence of specific mechanisms of its upregulation in response to light/oxidative conditions. In addition, our data demonstrate an overall positive effect of SkQ1 on antioxidant defense of the illuminated retina involving enhancement of total AOA and sustaining normal GPx activity.

### 3.4. Redox-Sensitive Proteins in Light-Induced Oxidative Stress: Visual Arrestin

**Figure 6:**
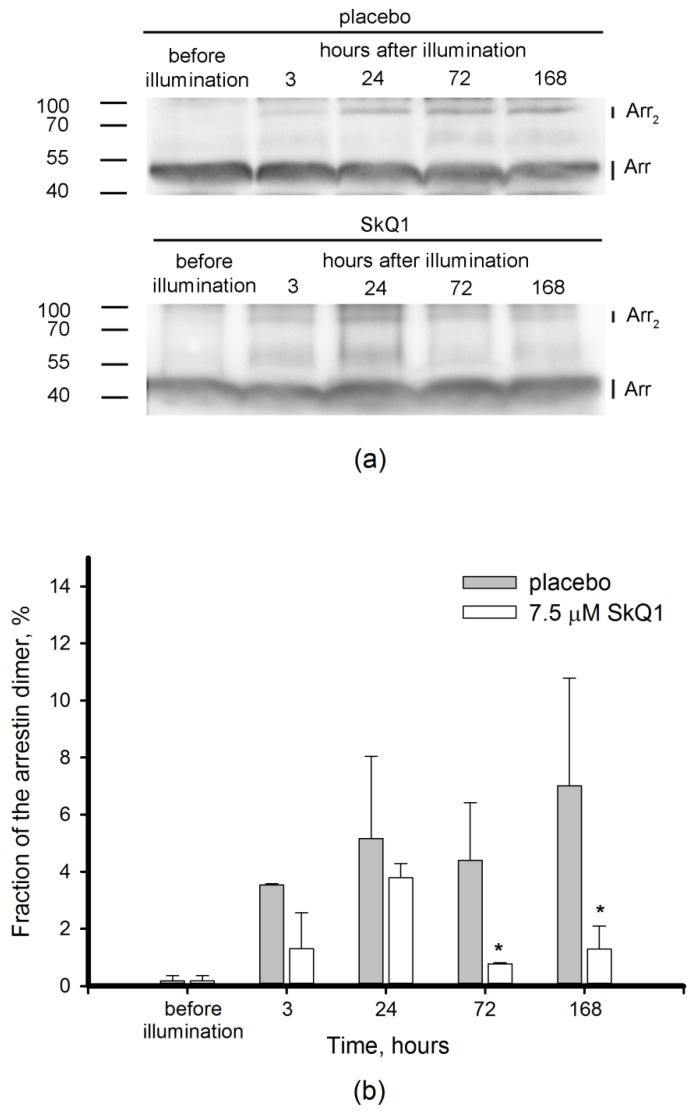
Monitoring of visual arrestin forms in extracts of intact and illuminated rabbit retinas with or without premedication with 7.5 μM SkQ1. (**a**) Western blotting of retinal extracts (normalized by total protein content) under non-reducing conditions using anti-visual arrestin antibodies; (**b**) weight fractions of disulfide dimers of arrestin determined from the Western blotting data. Each bar represents the data obtained from three animals (6 eyes). * *p* ≤ 0.05 compared with the values measured in the control animals.

Light-induced oxidative stress is known to be initially triggered in photoreceptor cells of the retina, which contain primary photosensitizing molecules [8]. To explore oxidative changes in photoreceptors in our model and to assess the effect of SkQ1 premedication on these changes, we examined the redox-state of visual arrestin, a crucial photoreceptor-specific protein possessing redox-sensitivity in vivo [20,41]. Particularly, we monitored the content of the disulfide dimers of arrestin in retinal extract by means of non-reducing Western blotting. In previous models, the maximal arrestin oxidation was observed within a few hours after the light exposure. With that in mind, in the arrestin experimental groups the animals were sacrificed before illumination or after 3, 24, 72 or 168 h after the exposure. As can be seen from Figure 6a, no band corresponding to arrestin dimer was observed in unexposed animals. Meanwhile, illumination triggered the formation of the dimer, which gradually accumulated in the retina within the course of the post-exposure period (Figure 6a). The band corresponding to arrestin dimer was absent in the Western blots performed under reducing conditions (data not shown), thereby confirming that arrestin dimer is stabilized by disulfide bonds. Remarkably, premedication with SkQ1 almost completely suppressed disulfide dimerization of arrestin (Figure 6b). The effect of the antioxidant was most noticeable late in the observation period, when the dimer content was 5.5-fold lower in SkQ1 premedicated animals as compared to the placebo group. Thus, premedication with SkQ1 protects visual arrestin from light-induced oxidation reflecting prominent inhibition of oxidative stress in photoreceptor cells.

## 4. Discussion

The main aim of this study was to characterize oxidative stress and intrinsic antioxidant responses in the retina exposed to bright light with or without premedication using SkQ1 in order to trial the potential of the antioxidant in prevention of oxidative processes contributing to photoreceptor loss. In our model, the illumination of rabbit eyes with visible light of high intensity (0.15 W/cm^2^, 3 h) induced almost a 20% decrease of ONL thickness over 7 days (Figure 1). Death of photoreceptors occurred simultaneously with the development of pronounced oxidative stress in the retina, which affected both the membranes and cytosol components of the retinal cells and resolved only at the seventh day after exposure. First, the illuminated retina exhibited increased lipid peroxidation manifesting in elevation of MDA content (Figure 2). These data are generally in agreement with the results obtained using similar animal models [42,43]. MDA is widely regarded as marker of light-induced oxidative stress in the illuminated retina. In RPE, MDA is produced by photoreactive lipofuscin granules, which also generate H_2_O_2_ and other potentially cytotoxic molecules in response to light [44]. Photoreceptor outer segments represent another major source of lipid oxidation products, due to high content of polyunsaturated fatty acids [45,46]. Remarkably, one hallmark of the dry (non-neovascular) form of AMD is the accumulation of debris in the form of large drusen within the Bruch’s membrane and/or RPE in the macular region and geographic atrophy of the RPE, followed by degeneration of the adjacent photoreceptor cells [6,47]. The proteins contained in the drusen of AMD patients were demonstrated to be modified by MDA [48]. Furthermore, they possess modifications by carboxyethyl pyrrole, induced by oxidation of docosahexaenoic acid, which originates mainly from photoreceptor membranes [49,50]. The latter observation supports the involvement of light-induced lipid damage in photoreceptor cells in AMD pathogenesis. 

Second, we report for the first time a dramatic increase in H_2_O_2_ concentration in extracts of the rabbit retinas exposed to short-term bright light (Figure 3). Previously, it was shown that progression of retinal damage induced by low-intensity light in rats is associated with H_2_O_2_ accumulation in photoreceptor cells [51]. Hydrogen peroxide is an important mediator of photoreceptor phototoxicity as indicated by efficacy of the specific H_2_O_2_ and hydroxyl radical scavengers for treatment of their light-induced damage in model animals [42,52,53]. Indeed, H_2_O_2_ is capable of oxidizing and consequently damaging photoreceptor lipids and proteins [8,20,48]. In RPE cells, H_2_O_2_ induces selective damage to mitochondrial DNA [26]. Interestingly, these cells display similar responses being incubated with hydrogen peroxide or preparations of photoreceptor outer segments [54]. This observation confirms H_2_O_2_ accumulation in photoreceptors, which makes their phagocytosis toxic to RPE cells by imposing a collateral oxidative burden in addition to intrinsic lipofuscin-dependent ROS generation [3,44]. 

Third, we revealed that the development of oxidative stress in the retina of the illuminated animals is associated with an imbalance of intrinsic antioxidants. Thus, light exposure stimulated AOA, but inhibited activity of antioxidant enzymes in the retina (Figure 4 and Figure 5). AOA encompasses activity of presumably low molecular weight antioxidants, commonly including glutathione, ascorbate, α-tocopherol, carotenoids, and flavonoids [55]. Among these compounds, glutathione and ascorbate are present in the retina in millimolar concentrations, providing the bulk of constitutive antioxidant activity in the tissue [56]. Unexpectedly, in our experiments the healthy retina was deprived of intrinsic AOA (Figure 4). In part, this effect could be attributed to limitations of the standardized luminescence assay [37]: even an unexposed retina contains relatively high amounts of ROS capable of oxidizing luminol and thereby interfering with AOA measurements [57]. We speculate that in intact retinas the intrinsic antioxidant components were engaged in scavenging ROS and the remainder was insufficient to noticeably inhibit luminol oxidation in the assay. In any case, our data indicate that AOA of healthy retina is considerably lower than in the other ocular tissues measured by the same method [34,38,39]. Meanwhile, the high-intensity light illumination clearly enhances AOA. These data suggest that oxidative stress might trigger a compensatory stimulation of the antioxidant defense. Indeed, prolonged light exposure was previously found to enhance ascorbate and α-tocopherol generation in rat retina [58]. 

AOA elevation was contrasted by a decrease in activity of antioxidant enzymes, SOD and GPx, after 3 days of the post-exposure period (Figure 5). In the retina, the same as in the other tissues, SOD converts superoxide anion into hydrogen peroxide, whereas GPx catalyzes its reduction by glutathione to form water [55,59]. Consistent with our data, SOD was found to be downregulated in the retina of rats and mice in response to light illumination [42,51]. Similarly, GPx activity decreased or unchanged in rat retinas underwent light-induced oxidative stress [42,60]. Interestingly, the long-term illumination contrariwise elevated GPx expression both in photoreceptors and RPE cells [60], suggesting that additional activity of the enzyme activity is required to suppress prolonged accumulation of ROS. Consistently, an increased level of GPx was observed in the blood plasma of AMD patients [55]. One can suppose that the increase in GPx represents one of the compensatory responses of the retina adapted to long-term oxidative stress. Indeed, improvement of baseline GPx activity was demonstrated in rats exposed to 800 lx light from birth to the age of 12 weeks [58]. Taken together, these data indicate that GPx malfunction contributes to light-induced ocular pathology and induction or compensation of its activity can be regarded as a promising approach for prevention and/or treatment of such conditions.

As it is described above, the oxidative biochemical changes in the retina exposed to intense light illumination involve mostly photoreceptors and RPE cells. Our experiments confirmed that oxidative stress engulfed photoreceptors as light exposure of the rabbit eyes induced disulfide dimerization of photoreceptor-specific protein, visual arrestin (Figure 6). Furthermore, we can anticipate that photooxidative processes are triggered in photoreceptor outer segments, as arrestin is known to translocate to these cellular compartments in response to rhodopsin activation by light [61]. Photoreceptor outer segments are believed to be the primary source of ROS in the retina due to the abundance of photosensitized reactions in these compartments. Indeed, light-dependent accumulation of all-*trans*-retinal in photoreceptor discs yields generation of superoxide anion, singlet oxygen, and organic peroxides [8,22,62]. Superoxide anion is regarded as one of the key mediators of LIRD [24]. SOD and ascorbate convert correspondingly superoxide anion and singlet oxygen into hydrogen peroxide [63]. Consistently, the level of H_2_O_2_ in photoreceptors increases in response to intensive light illumination (Figure 3, [51]). Hydrogen peroxide is known to primarily affect the cysteines in proteins, resulting in oxidation of thiolate to form sulfenic acid. The latter interacts with the adjacent cysteines yielding intramolecular or intermolecular disulfides. Otherwise it can be further oxidized to sulfinic or sulfonic acid [64,65]. Arrestin is one of the photoreceptor proteins responding to oxidative stress in the retina by forming disulfide dimers under in vivo conditions [20,41]. In the current study, we found that arrestin dimers were formed in the retina as early as three hours post exposure and they accumulated over time, reaching a maximum on day 7, when the oxidative stress ceased and H_2_O_2_ levels normalized (Figure 2 and Figure 3). Thus, arrestin can be regarded as an early and stable marker of light-induced oxidative stress in photoreceptors.

Photoreceptor cells are characterized with a high metabolic rate [1]. As a result, light-induced oxidative stress in the photoreceptor outer segments is aggravated by constant ROS generation in the mitochondria. Indeed, these organelles are involved in mechanisms of oxidative damage to photoreceptors under conditions of excessive illumination by visual light [24]. Furthermore, mitochondria are important mediators of light-dependent oxidative damage to RPE cells [25,26]. Considering these observations, we proposed that LIRD can be effectively prevented by mitochondria-targeted antioxidants. Indeed, our data demonstrated that SkQ1 prevented photoreceptor cell loss (Figure 1) and prominently improved redox status of the illuminated retina. Premedication of the rabbit eyes with SkQ1 alleviated oxidative stress in this tissue (Figure 2): it inhibited oxidation of membrane components and suppressed generation of H_2_O_2_ responsible for oxidation of retinal proteins (Figure 3). Consistently, pre-treatment with SkQ1 prevented light-induced disulfide dimerization of arrestin (Figure 6), indicating suppression of oxidative stress in the photoreceptors. Furthermore, antioxidant premedication produced a long-term enhancing effect on AOA in light-exposed retinas (Figure 4) and delayed the downregulation of GPx activity in response to oxidative stress (Figure 5b). 

The ability of SkQ1 to suppress oxidative stress in ocular tissues was demonstrated in previous studies [19,32]. The antioxidant possesses high bioavailability upon topical administration in the form of eye drops and exhibits prominent protector activity towards different eye tissues [32,33,34]. Meanwhile, our data represent the first direct evidence of the antioxidant effect of SkQ1 in the retina. We propose that SkQ1 suppresses accumulation of hydrogen peroxide and lipid oxidation via prevention of ROS generation in the inner segment of photoreceptors, i.e. in the place impregnated with mitochondria. This effect facilitates neutralization of ROS, the products of photosensitized reactions in outer segments, by the components of intrinsic antioxidant defense. The suppression of ROS production might additionally release AOA components thereby enhancing their compensatory rise in response to light. Meanwhile, the effect of SkQ1 premedication on the activity of major antioxidant enzymes was moderate and divergent. Thus, the antioxidant had no reliable effect on SOD activity, whereas GPx activity was likely delayed in the premedicated animals as compared to control. Slight improvement of GPx activity by SkQ1 was previously described for other tissues [34,66]. In any case, topical premedication of the eyes with SkQ1 apparently could help to sustain normal activity of GPx in the retina under conditions of light-induced oxidative stress. Since oxidative stress together with GPx malfunction highly contributes to pathogenesis of ophthalmological disorders, specifically AMD (see above) [55], one can expect efficacy of SkQ1 in prophylaxis of the disease. 

The high potential of SkQ1 for ocular protection is further evidenced from its ability to completely inhibit disulfide dimerization of photoreceptor-specific redox-sensitive protein arrestin. Oxidation of this protein was speculated to disrupt its function, which consists in shutting down light-activated rhodopsin [20,41]. Arrestin malfunction is expected to accelerate accumulation of *all-trans*-retinal and its light-sensitive toxic metabolites (retinoids) in photoreceptor discs under conditions of excessive light stimuli [67,68,69,70]. Photoreceptor discs are known to be recycled by the RPE cells, which would thereby be exposed to retinoid toxicity. Similar processes were reported to contribute to pathogenesis of light-induced retinopathy and AMD [71]. Consistently, arrestin-deficient animals exhibit lower resistance to retinal damage induced by visible light [72]. Considering these putative effects of arrestin oxidation, the protection of its redox status by SkQ1 might improve the resistance of the retina to light- and age-related damage.

It should be added that AMD drusen likely contain the components of photoreceptor outer segments consumed and incompletely digested by the RPE [73]. Some of the drusen proteins have oxidative modifications, indicative of their exposure to oxidative stress. The ability of SkQ1 to prevent disulfide dimerization of visual arrestin reflects its high efficacy in targeting oxidative stress in photoreceptor cells and maintaining normal redox-status of photoreceptor proteins. Given that the major oxidative processes triggering AMD progression originate from photoreceptors [24], the prompt application of SkQ1 might suppress early phases of the disease. Thus, this condition can be added to the list of age-related ocular diseases responsive to premedication and treatment with SkQ1 [32,33]. 

## 5. Conclusions

Exposure to 30,000 lx visible light for 3 h induced pronounced oxidative stress in the retina, manifesting in lipid peroxidation, H_2_O_2_ generation, and decrease of antioxidant enzyme activity, contrasted by compensatory growth of low molecular weight antioxidants (AOA). Photoreceptor cells were highly susceptible to these light-induced changes, as indicated by ONL thinning and oxidation of photoreceptor-specific protein arrestin, which formed disulfide dimers as an early response to the irradiation. Mitochondria-targeted antioxidant SkQ1 was demonstrated for the first time to prevent light-induced retinal/photoreceptor damage. Premedication with SkQ1 suppressed lipid and protein oxidation, as well as stimulated innate antioxidant defense of the tissue. Overall, our data suggest that SkQ1 can be employed for prevention of light-induced (including iatrogenic) retinopathies, AMD and other retinal degenerative diseases.

## Abbreviations

AMD	Age-related macular degeneration
RPE	Retinal pigment epithelium
ROS	Reactive oxygen species
LIRD	Light-induced retinal damage
SkQ1	10-(6′-plastoquinonyl)-decyltriphenylphosphonium
AOA	Total antioxidant activity
SOD	Superoxide dismutase
GPx	Glutathione peroxidase
PBS	Phosphate buffer saline
